# Special Issue: Retroviral Enzymes

**DOI:** 10.3390/v2051181

**Published:** 2010-05-07

**Authors:** Luis Menéndez-Arias

**Affiliations:** Centro de Biología Molecular “Severo Ochoa” [Consejo Superior de Investigaciones Científicas (CSIC) & Universidad Autónoma de Madrid], Campus de Cantoblanco, 28049 Madrid, Spain; E-Mail: lmenendez@cbm.uam.es; Tel.: +34 91 196 4494; Fax: +34 91 196 4420

The retroviral RNA genome encodes for three enzymes essential for virus replication: (i) the viral protease (PR), that converts the immature virion into a mature virus through the cleavage of precursor polypeptides; (ii) the reverse transcriptase (RT), responsible for the conversion of the single-stranded genomic RNA into double-stranded proviral DNA; and (iii) the integrase (IN) that inserts the proviral DNA into the host cell genome. All of them are important targets for therapeutic intervention. This Special Issue provides authoritative reviews on the most recent research towards a better understanding of structure-function relationships in retroviral enzymes. The Issue includes three reviews on retroviral PRs, seven on RT and reverse transcription, and four dedicated to viral integration.

The viral PR plays a critical role at the last stage of viral replication by processing of Gag and Gag-derived polyproteins at a limited number of sites. Detailed analysis of the substrate specificity of retroviral PRs reveals the existence of two types of cleavage sites with different specificities. However, it is not possible to give a consensus substrate sequence, based on known processing sites. Substrate specificity in retroviral PRs is addressed in the review by Tözsér [1]. Understanding the specificity of these enzymes should be helpful to design broad-spectrum inhibitors targeting human immunodeficiency virus type 1 (HIV-1) and other retroviruses.

The approval of first-generation HIV-1 PR inhibitors such as saquinavir led to the introduction of highly active antiretroviral therapy (HAART) in 1995. HAART has been a revolutionary treatment towards the control of AIDS. However, antiviral drug resistance, deriving from viral replication mutability, and also the high price of PR inhibitors and problems of tolerability, toxicity and tolerance has triggered further research on HIV PRs. Second generation inhibitors designed to inhibit PRs resistant to first generation inhibitors have been developed to minimize side effects and improve dosing. Examples are lopinavir, atazanavir, tipranavir and darunavir. Developments in this area, as well as descriptions of novel inhibitors in the pipeline, such as PL-100, brecanavir and GS 8374, and others targeting PR dimerization or the flaps are discussed in the review by Pokorná *et al*. [2].

The molecular mechanisms associated with PR inhibitor resistance, mediated by mutations that alter PR interactions with inhibitors or substrates, or dimer stability; as well as distal mutations that transmit changes to the active site of the enzyme are discussed in the light of the structural information available in the review authored by Weber and Agniswamy [3]. These authors show how structural data have provided guidelines towards the design of novel HIV PR inhibitors, such as GRL-02031 based on darunavir. In those studies, it has been shown that the formation of hydrogen bonds between the drug and the backbone of the PR can be a valuable strategy for improving resilience to drug resistance, by favouring minimal changes in the PR backbone between wild-type and mutant enzymes [3].

The reverse transcription of the viral single-stranded (+) RNA genome into double-stranded DNA (dsDNA) is an essential step in retroviral replication, and therefore an important target for therapeutic intervention. Retroviral RTs have two distinct activities: (i) a DNA polymerase activity which uses either RNA or DNA as template; and (ii) an RNase H activity, which degrades RNA from RNA/DNA hybrids. HIV-1 RT is a heterodimer composed of two subunits of 66 kDa and 51 kDa, and the major target of anti-AIDS therapies. Singh *et al.* [4] provide a detailed account of the crystallographic work leading to our current knowledge of the HIV-1 RT structure and its mechanism of action. Their review focuses on the structural basis of RT inhibition by nucleoside and nonnucleoside RT inhibitors, with special attention to the effects of drug resistance mutations.

Retroviral RTs are devoid of 3′→5′ exonucleolytic proofreading activity and their mutation rates are around 10^−4^ to 10^−5^, well above the values reported for cellular DNA polymerases. Their contribution to mutagenesis, and therefore to the emergence of drug resistance is discussed in a review on the intrinsic fidelity of retroviral RTs [5]. In this review, the author provides an update on the molecular basis of fidelity of HIV-1 RT, based on published data obtained *in vitro* by using different methods, based either on the expression of genes such as *lacZ* or in measurements of nucleotide selectivity (*e.g*., the incorporation of correct *versus* incorrect nucleotides).

Reverse transcription is a relatively complex process (reviewed in [6]) that initiates after binding of a specific cellular tRNA to the primer binding site (PBS) located in the 5′-end of the viral genome. The RT uses the tRNA as a primer and copies the 5′-end of the RNA genome in the so-called first (-) strand DNA synthesis. The synthesized RNA/DNA hybrid is degraded by the RNase H activity of the RT to generate the (-) strand single-stranded DNA (ssDNA). Reverse transcription initiation emerges as a distinct process with intervention of tRNA and viral proteins such as the nucleocapsid (NC) protein or Vif. In their review, Isel *et al*. [7] provide an update on our current understanding of the formation of the initiation complex of HIV-1 reverse transcription, paying special attention to the spatio-temporal regulation of the process and the participation of viral cofactors.

The sequences at the 5′- and 3′-ends of the viral RNA genome are identical, and this characteristic allows the (-) strand ssDNA to hybridize with the 3′-end of the viral RNA. As DNA synthesis continues, RNase H degrades the RNA strand. A specific sequence, rich in purines (termed as polypurine tract or PPT) near the 3′-end of the viral RNA is relatively resistant to cleavage by RNase H and serves as primer for the synthesis of the second (+) strand of the DNA. Synthesis of (+) strand viral DNA is a tightly regulated process that can be reproduced in the laboratory in the absence of accessory viral and host factors. The communication between RT and the RNA/DNA hybrid is necessary to promote these events. The interaction between RT and its cognate PPT has been studied by using sophisticated biophysical techniques (*e.g*., chemical probing, mass spectrometry, NMR spectroscopy, and single molecule spectroscopy), and the obtained results are discussed in the review by Fabris *et al*. [8]. Synthesis of (+) strand DNA is followed by removal of the tRNA by the RNase H activity of the RT. A second transfer event, followed by extension of (+) and (-) strands leads to completion of proviral DNA synthesis. At both ends, proviral DNAs contain the same sequence (designated as long terminal repeats or LTRs).

The role of the HIV-1 RNase H activity in reverse transcription, its structure and catalytic mechanisms, and potential ways to inhibit the enzyme are discussed in the review by Beilhartz and Götte [9]. Since none of the approved anti-HIV agents inhibit specifically the RNase H function of RT, the development of novel and potent antiviral agents targeting RNase H remains as a challenging opportunity for future research.

Synthesis of proviral DNA is catalyzed by the viral RT, but other viral proteins such as NC play an important role as nucleic acid chaperones, during the strand transfers required to complete viral DNA synthesis [6]. Recent studies suggest that HIV-1 uses hundreds of cellular factors during the virus life cycle. The involvement of cellular proteins in the regulation of reverse transcription is discussed in the review by Warren *et al*. [10]. Cellular proteins such as HuR, AKAP149 and DNA topoisomerase I have been shown to interact with HIV-1 RT. In addition, Gemin2 and components of the Sin3a complex can also affect reverse transcription, through the interaction of the RT with the viral IN.

Integration of the proviral DNA into the host chromosome is an essential step in the retroviral life cycle. The viral IN recognizes the two viral DNA ends and through relevant 3′-processing and strand transfer activities completes the integration reaction. In the review by Kessl *et al*. [11], the catalytic properties and structure of HIV-1 IN are discussed in the context of its interactions with the DNA, paying special attention to current research on the oligomeric forms of the enzyme, the role of its principal cofactor in HIV-1 and other retroviruses (*i.e*., lens epithelium derived growth factor, LEDGF/p75), and the characterization of relevant nucleoprotein complexes containing viral IN. Structural aspects of the interaction between LEDGF and lentiviral INs are specifically addressed by Hare and Cherepanov [12]. Several studies discussed in their review emphasize on the characteristic preference of lentiviruses to integrate within transcription units. It is now clear that the chromosomal site of viral integration is not random, and retroviruses display specific preferences at distinct genomic positions. Desfarges and Ciuffi [13] summarize current knowledge on retroviral integration site preferences and the mechanisms involved in the process. Again, a prominent role is given for LEDGF/p75, in combination with histone methyltransferases associated with active transcription.

The last review of the Issue focuses on the contribution of host proteins to viral integration in the murine leukaemia virus (MLV) [14]. The functional equivalent of LEDGF/p75 in MLV is not known, perhaps reflecting a different nuclear entry mechanism used by HIV-1 *versus* MLV. However, there are many host factors that have been implicated in interactions with MLV pre-integration complexes. Examples include *bona-fide* and putative transcription factors (*e.g*., TFIIE-β, Ankrd49, Znfp38, ABT1, *etc.*), endonucleases (*e.g*., Fen1) or repair proteins (*e.g*., Ku70/XRCC6), among others. A better knowledge of MLV integration could be helpful to understand oncogene activation, and for designing better vectors to be used in gene therapy.

I hope that this collection of reviews will contribute to a better knowledge of the field, while encouraging research and promoting interest in retroviral enzymes among graduate students and young scientists. Finally, I would like to thank authors for their valuable contributions and all reviewers for their constructive critiques.
